# Etymologia: *Culex quinquefasciatus*

**DOI:** 10.3201/eid2708.ET2708

**Published:** 2021-08

**Authors:** Sarah Anne J. Guagliardo, Rebecca S. Levine

**Affiliations:** Centers for Disease Control and Prevention, Atlanta, Georgia, USA

**Keywords:** Culex quinquefasciatus, mosquitoes, mosquito vectors, Culicidae, vector-borne infections

## *Culex quinquefasciatus* [′kyo͞o leks ′kwinkwə fa she ′ah tus]

In 1823, the American entomologist Thomas Say described *Culex* (Latin for “gnat”) *quinquefasciatus*, which he collected along the Mississippi River. Originally written as “C. 5-fasciatus,” the name refers to 5 (“quinque”) black, broad, transverse bands (“fasciatus” or “fasciae”) on the mosquito’s dorsal abdomen (Figure). The name remains despite later revelations of more than 5 fasciae, thanks to improved microscopy. Although *quinquefasciatus* is the official scientific name, there are at least 5 synonymous names for this species.

**Figure:**
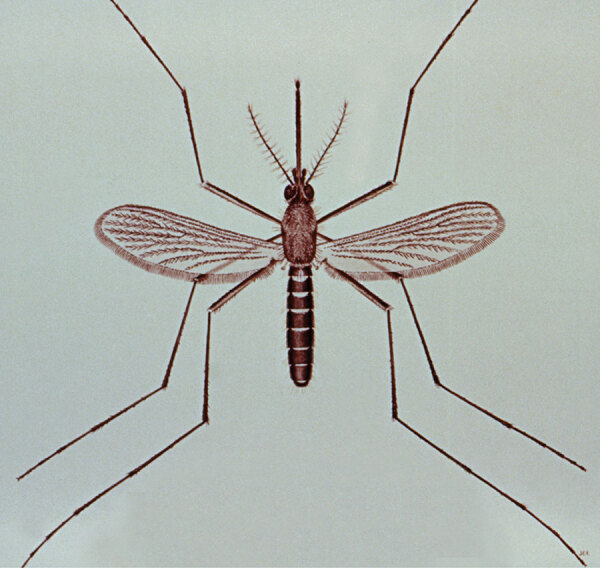
Female *Culex quinquefasciatus* mosquito. Image credit: CDC Public Health Image Library, 1976.)

Say described this species as “exceedingly numerous and troublesome.” “Quinx” are among the world’s most abundant peridomestic mosquitoes, earning the nickname “southern house mosquito.” *Cx. quinquefasciatus* is found throughout subtropical and tropical areas worldwide, except for exceedingly dry or cold regions. This mosquito is a principal vector of many pathogens, transmitting the phlebovirus Rift Valley fever virus and the 2 flaviviruses St. Louis encephalitis virus and West Nile virus, in addition to filarial worms and avian malarial parasites.
